# Early positive signals of cognitive outcomes in mild to moderate Alzheimer patients treated with the synaptic regenerative small molecule, SPG302

**DOI:** 10.1002/alz70861_109023

**Published:** 2025-12-23

**Authors:** Lauren Priest, Aimee Cayzer, Bruce Brew, Valerie Bramah, Craig Erickson, Peter Vanderklish, Sharron Gargosky, Stella Sarraf

**Affiliations:** ^1^ Flinders Medical Centre, Adelaide, SA Australia; ^2^ St Vincent’s Centre for Applied Medical Research, Sydney, NSW Australia; ^3^ St Vincent's Hospital, Melbourne, VIC Australia; ^4^ Spinogenix Inc, Los Angeles, CA USA

## Abstract

**Background:**

Alzheimer’s disease (AD) is characterized by an early and progressive loss of glutamatergic synapses, a core feature of the disease that contributes to impairments in memory and cognition. Synaptic regeneration has long been held as a potentially viable approach to slowing or even reversing memory and cognitive symptoms, a hypothesis supported by the observation that aged individuals with normal synaptic density demonstrate cognitive resilience in the face of AD‐like molecular pathology.

SPG302 is a novel, rapid‐acting synaptogenic small molecule developed by Spinogenix. In preclinical studies using a mouse model of AD (Trujillo‐Estrada et al, 2021), once‐daily treatment with SPG302 for 1 month rapidly reversed large deficits in axospinous glutamatergic synapses in the hippocampus and performance on hippocampal dependent tasks. Phase 1 trials in healthy volunteers established a favorable safety and tolerability profile and dose‐dependent plasma exposures consistent with efficacy in preclinical models. The safety and potential efficacy of SPG302 in AD is currently being evaluated in a Phase 2 trial, the first cohort of which is complete.

**Method:**

Spinogenix initiated a Phase 2, multicenter study in Australia to assess the safety, tolerability, pharmacokinetics (PK), pharmacodynamics and clinical efficacy of SPG302 in adult participants with mild‐to‐moderate AD. An initial cohort of 12 AD participants received 300mg SPG302 or placebo (2:1 randomization) daily as an oral tablet for a period of 4 weeks, followed by open‐label extension for an additional 24 weeks. Outcomes, assessed on a monthly basis, included standard cognitive assessments (MMSE, CDR‐sb, ADAS‐cog), quantitative neurophysiological measures sensitive to synaptic density (resting state EEG and auditory P300), quality of life and activities of daily living measures, and other measures.

**Result:**

SPG302 was safe and well tolerated. Rapid improvements in MMSE were observed in most patients, with corresponding changes in other cognitive measures. PK parameters in AD subjects were similar to healthy controls. These data, and EEG measures under analysis at the time of submission, will be presented.

**Conclusion:**

Once‐daily treatment with the novel synaptogenic small molecule, SPG302, is safe and well tolerated in AD participants over the course of 28 weeks was associated with early signs of cognitive improvements.

